# The association between race and risk of illness and death due to COVID-19: A protocol for systematic review and meta-analysis

**DOI:** 10.1097/MD.0000000000022828

**Published:** 2020-11-13

**Authors:** Talita Araujo de Souza, Pedro Henrique Alcântara da Silva, Aryelly Dayane da Silva Nunes, Ivani Iasmim de Araújo, Victor Hugo de Oliveira Segundo, Dalyanna Mildred de Oliveira Viana Pereira, Isabelle Ribeiro Barbosa, Gilson de Vasconcelos Torres

**Affiliations:** a Postgraduate Program in Health Sciences; b Postgraduate Program in Public Health; c Faculty of Health Science of Trairi, Federal University of Rio Grande do Norte, Santa Cruz, RN, Brazil.

**Keywords:** Covid-19, severe acute respiratory syndrome coronavirus 2, African Americans, race

## Abstract

**Background:**

The Corona Virus Disease, 2019 (COVID-19) pandemic revealed many social disparities that already exist in countries that have social inequalities in their historical context. Studies have already been published on the epidemiological and clinical characteristics of population groups considered to be at risk where they reveal that Black people are at greater risk of becoming ill and dying from this cause. In this context, this protocol describes a systematic review that aims to analyze the association of race as the higher risk for illness and death due to COVID-19.

**Methods:**

This protocol will be developed based on the recommendations of Preferred Reporting Items for Systematic Reviews and Meta-Analyses (PRISMA-P). For this, we will conduct searches in the PubMed, Web of Science, Scopus, Lilacs, and ScienceDirect databases in the search for cross-sectional studies. All cross-sectional studies that analyzed hospitalization and death by COVID-19 as race in its determinant will be included. The search will be carried out by 2 independent researchers who will carry out the selection of articles, then the duplicate studies will be removed and screened using the Rayyan QCRI application. To assess the risk of bias, the instrument proposed by Downs and Black will be used. Meta-analyzes and subgroup analyzes will be carried out according to included data conditions.

**Results:**

Based on this review, it will be possible to carry out a high-quality synthesis of available evidence that brings race as a factor for illness and death by COVID-19 and to verify which race is most affected by this disease.

**Conclusion:**

The relevance of this systematic review to the current context is considered, as it has a high potential to assist in the development of public health strategies and policies that address existing racial differences.

Record of systematic review: CRD42020208767.

## 1 Introduction

The Corona Virus Disease, 2019 (Covid-19) pandemic has devastated the global community. As of September 14, 2020, there are 29.075.608 in the world and Brazil occupies the third place with the highest number of confirmed cases, totaling 4,330,455^[1]^ and second place with the highest number of deaths with 131,625 so far.^[1]^ This pandemic is seen as a challenge for countries that have deep historical social inequalities.^[2]^ In this scenario, the deaths by COVID-19 in places like New York and other metropolitan areas of the United States, once again accentuated the racial/ethnic issue to health disparities, including comorbidities associated to COVID-19.^[3]^

Research already shows that hospitalizations and deaths due to COVID-19 are unevenly distributed by race and ethnicity, where Black individuals comprised 35% of hospitalizations due to COVID-19 and 31% of deaths in New York.^[4]^ The differences that are seen in the hospitalization and mortality rates due to COVID-19 reflect the general disparity trends in racial / ethnic health, which emerge from interactions of ecological variables, such as poverty and access to health and individual factors, such as chronic diseases.^[5]^

Studies show that Black people have a higher prevalence of comorbidities such as hypertension and diabetes.^[6,7]^ This association between Black color and higher prevalence of hypertension and diabetes is explained by unfavorable sociodemographic characteristics, such as income, education and health insurance, and not by color.^[6]^ However, these comorbidities are considered to be risk factors for COVID-19,^[8]^ and may therefore present a higher risk of serious cases and deaths in this population. Based on this, we want to analyze whether the clinical characteristics of patients with COVID-19 differ between races and whether these characteristics are associated with risks of illness and death.

## 2 Methods and analysis

### 2.1 Protocol and registration

This systematic review was recorded in the International prospective register of Systematic reviews (PROSPERO) on Sep 14, 2020 under the number CRD42020208767. Available at: https://www.crd.york.ac.uk/prospero/display_record.php?ID=CRD42020208767.

### 2.2 Selection process

**Figure 1:**
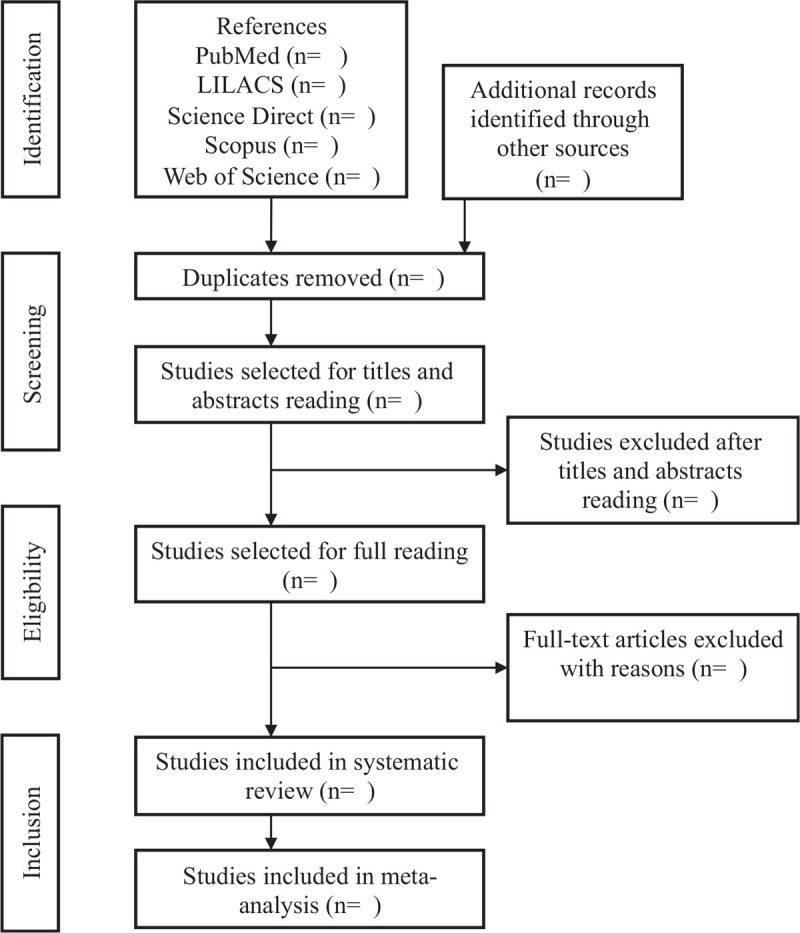
Flow diagram. Adapted from PRISMA-P.

The design and development of this systematic review and meta-analysis will be in accordance with the statement of Preferred Reporting Items for Systematic Reviews and Meta-Analyses (PRISMA-P).^[9]^ Initially, the collection of bibliographic data will be made in the electronic databases: PubMed, Lilacs, Web of Science, Scopus, and Science Direct. In order to carry out the appropriate search in each database, the search strategy will be duly modified for each one and will be carried out by 2 reviewers in a double-blind manner to identify the eligible studies. This pair of independent researchers will carry out the search, and publications considered to be potentially relevant will be included in the review if they meet all the inclusion criteria. Consensus meetings will be held at each stage, if there is no consensus the third reviewer will participate. Cohort studies, case control, case reports, systematic reviews, randomized clinical trials, and qualitative studies will be excluded. The reference list of possible studies included will be selected manually to identify other relevant publications. In case of disagreement, it will be resolved by a third reviewer (Fig. 1).

### 2.3 Search strategy

The search strategies will be used from the descriptors and keywords selected for the research, considering the following terms: (“Covid-19” [Mesh] or “COVID19” or “COVID-19 pandemic” or “SARS-CoV- 2 infection” ’or “COVID-19 virus disease”) or (“severe acute respiratory syndrome coronavirus 2” [Mesh] or “SARS-CoV-2” or “2019 novel coronavirus” or “COVID-19 virus”) and (“African Americans” [Mesh] or “African-Americans” or “African-American”) or (“Race” [Mesh]).

The Rayyan QCRI Software^[10]^ which is used for systematic reviews will be used to read the titles and abstracts, remove duplicate articles and read the full texts. For formatting references, we will use the Mendeley Software.^[11]^ Thus, during the process, the title and abstract will be read, then the duplicates will be removed, after which we will read the selected articles in full. If no abstract is provided from this search strategy, the full text can be reviewed and evaluated. If 2 independent researchers do not agree with the inclusion of any study in the review, the third researcher will decide whether or not to include that study.

### 2.4 Inclusion criteria

To prepare for this review, we will include all cross-sectional studies that analyzed the admission and death of patients with COVID-19 cross-sectional studies that analyzed the admission and death of patients due to COVID-19 by race, considering the eligibility criteria adopted in the population of the study, Intervention, Comparison, Result and Study Design (PICOS), according to the details presented in Table 1.

**Table 1:** PICO description.

PICO	Abbreviation	Elements
**P**articipants	P	Black individuals
**E**xposition	E	People with COVID-19
**C**omparison	C	People with COVID-19 of other races
**O**utcome	O	Risk of illness and death due to COVID-19

### 2.5 Exclusion criteria

We will exclude studies according to the following criteria: Studies that do not present the race variable in their results as a social determinant; Studies such as cohort, case control, case reports, reviews and Randomized Clinical Trials, and qualitative studies

### 2.6 Data collection process

The characteristics of the study (author, publication date, study design, period, race) and study population (study location, sex, and age group of participants) will be extracted from all included studies. We will identify peer-reviewed publications that include the following criteria: Black individuals (participants); people with COVID-19 (exhibition); people with COVID-19 from other racial / ethnic groups (control); Risk of illness and death due to COVID-19 (outcome). Two reviewers will extract data from all articles independently, and any disagreement will be resolved by a third author.

### 2.7 Risk of bias assessment

To conduct an appraisal of the studies’ methodological quality, the included articles will be evaluated and score accord the quality index for randomized and observational studies proposed by Downs and Black.^[12]^ Each published paper will be evaluated independently by 2 authors. To settle any disagreements in assigned scores, a third author will be consulted. The quality index is a 26-item checklist including 5 subscales: reporting; external validity; internal validity—bias; internal validity—confounding; and power. Items are scored 0 or 1, except for 1 item in the reporting subscale, scored 0 to 2, and the single power item, scored 0 to 5. The total maximum score for quality is 32.

### 2.8 Data synthesis

Results will be expressed as odds ratios with 95% confidence intervals. Fixed-effects or random effects models will be chosen depending on whether there is an absence or presence of heterogeneity between studies. Statistical heterogeneity will be assessed by the I2 statistic (<25%, no heterogeneity; 25%–50%, moderate heterogeneity; and >50%, strong heterogeneity). When a significant heterogeneity exists across the included studies (*I*^2^ > 50%), a random-effects model will be used for the analysis; otherwise, the fixed-effects model will be used. We will use the Egger funnel plot to assess possible publication bias. All tests will be performed using Review Manager (RevMan version 5.3.0) software and 2-sided *P* value <.05 will be considered statistically significant.

### 2.9 Confidence in cumulative evidence

The grading of recommendations, assessment, development, and evaluation (GRADE) approach will be used to assess the quality of evidence that will be included in this review

### 2.10 Ethics and dissemination

For the development of this study, approval of ethics and consent is not necessary because it is a systematic review that will use secondary studies.

## 3 Discussion

The COVID-19 pandemic has considerably impacted all health care sectors globally, revealing the profound inequalities in access and care to health services. Within this pandemic, it is possible to highlight the equity gap for marginalized groups in the face of society, especially for the Black community, which, as noted, is the population that has shown the greatest increase in the morbidity and mortality indicators due to COVID-19 when compared the population of other races.^[13]^

Analyzing individually, the risk factors already reported associated with hospitalization and death by COVID-19 is the presence of comorbidities such as hypertension, diabetes, heart disease in parallel with age.^[14–16]^ Black people have a higher prevalence of these comorbidities, this being one of the factors with the highest prevalence of COVID-19 in this group, however, researchers also associate the differences observed in the hospitalization and mortality rates of COVID-19 as a reflection of the general trends in racial / ethnic health disparities, which arise from interactions complex ecological variables, such as poverty and access to health and individual factors, in addition to chronic diseases.^[5]^

In the United States, primary studies already show these results as a reflection of racial disparities, in which African Americans are overrepresented in the scenario of illness and death due to the new coronavirus. In Michigan, African Americans are 14% of the population, they represent more than 30% of COVID-19 positive cases and more than 40% of deaths. In Chicago, Afro-Americans - 29% of the city's population – represent 70% of deaths by Covid-19.^[17,18]^

We hope that the completed systematic review will bring results that make us understand the main clinical characteristics of COVID-19 infection in different races and identify whether racial disparities are factors that may increase the risk of illness and death. In addition, the results can also assist in the definition of strategies that can be adopted for the control and prevention of COVID-19 transmission, to define pandemic and post-pandemic recommendations for health care and self-care.

## Author contributions

**Conceptualization:** Talita Araujo de Souza, Pedro Henrique Alcântara da Silva, Aryelly Dayane da Silva Nunes, Ivani Iasmin de Araújo, Victor Hugo de Oliveira Segundo, Isabelle Ribeiro Barbosa.

**Data curation:** Talita Araujo de Souza, Pedro Henrique Alcântara da Silva, Aryelly Dayane da Silva Nunes, Victor Hugo de Oliveira Segundo, Isabelle Ribeiro Barbosa.

**Formal analysis:** Talita Araujo de Souza, Pedro Henrique Alcântara da Silva, Victor Hugo de Oliveira Segundo, Isabelle Ribeiro Barbosa, Gilson de Vasconcelos Torres.

**Investigation:** Talita Araujo de Souza, Pedro Henrique Alcântara da Silva, Victor Hugo de Oliveira Segundo, Isabelle Ribeiro Barbosa.

**Methodology:** Talita Araujo de Souza, Pedro Henrique Alcântara da Silva, Victor Hugo de Oliveira Segundo, Isabelle Ribeiro Barbosa.

**Project administration:** Talita Araujo de Souza, Isabelle Ribeiro Barbosa.

**Resources:** Talita Araujo de Souza.

**Supervision:** Aryelly Dayane da Silva Nunes, Victor Hugo de Oliveira Segundo, Isabelle Ribeiro Barbosa, Gilson de Vasconcelos Torres.

**Validation:** Isabelle Ribeiro Barbosa.

**Visualization:** Aryelly Dayane da Silva Nunes, Isabelle Ribeiro Barbosa.

**Writing – original draft:** Talita Araujo de Souza, Ivani Iasmin de Araújo, Victor Hugo de Oliveira Segundo, Dalyanna Mildred de Oliveira Viana Pereira, Isabelle Ribeiro Barbosa.

**Writing – review & editing:** Talita Araujo de Souza, Pedro Henrique Alcântara da Silva, Ivani Iasmin de Araújo, Isabelle Ribeiro Barbosa, Gilson de Vasconcelos Torres.
